# Community health worker promotions increase uptake of long-acting reversible contraception in Rwanda

**DOI:** 10.1186/s12978-019-0739-0

**Published:** 2019-06-04

**Authors:** Amelia Mazzei, Rosine Ingabire, Jeannine Mukamuyango, Julien Nyombayire, Robertine Sinabamenye, Roger Bayingana, Rachel Parker, Amanda Tichacek, Sarah Rae Easter, Etienne Karita, Susan Allen, Kristin M. Wall

**Affiliations:** 1 Projet San Francisco, Rwanda Zambia HIV Research Group, Department of Pathology & Laboratory Medicine, School of Medicine, Emory University, Kigali, Rwanda; 2 Rwanda Zambia HIV Research Group, Department of Pathology & Laboratory Medicine, School of Medicine, Emory University, Atlanta, GA 30322 USA; 3 Division of Maternal-Fetal Medicine, Brigham and Women’s Hospital, Harvard Medical School, Boston, MA 02115 USA; 4 Department of Epidemiology, Rollins School of Public Health, Laney Graduate School, Emory University, Atlanta, GA 30322 USA

**Keywords:** Family planning, Contraception, Long-acting reversible contraception, Couples, Rwanda

## Abstract

**Background:**

We coordinated community health worker (CHW) promotions with training and support of government clinic nurses to increase uptake of long-acting reversible contraception (LARC), specifically the copper intrauterine device (IUD) and the hormonal implant, in Kigali, Rwanda.

**Methods:**

From August 2015 to September 2016, CHW provided fertility goal-based family planning counseling focused on LARC methods, engaged couples in family planning counseling, and provided written referrals to clients expressing interest in LARC methods. Simultaneously, we provided didactic and practical training to clinic nurses on LARC insertion and removal. We evaluated: 1) aggregate pre- versus post-implementation LARC uptake as a function of CHW promotions, and 2) demographic factors associated with LARC uptake among women responding to CHW referrals.

**Results:**

7712 referrals were delivered by 184 CHW affiliated with eight government clinics resulting in 6072 family planning clinic visits (79% referral uptake). 95% of clinic visits resulted in LARC uptake (16% copper IUD, 79% hormonal implant). The monthly average for IUD insertions doubled from 29 prior to service implementation to 61 after (*p* < 0.0001), and the monthly average for implant insertions increased from 109 to 309 (p < 0.0001). In adjusted analyses, LARC uptake was associated (*p* < 0.05) with the CHW referral being issued to the couple (versus the woman alone, adjusted odds ratio, aOR = 2.6), having more children (aOR = 1.3), desiring more children (aOR = 0.8), and having a religious affiliation (aOR = 2.9 Protestant, aOR = 3.1 Catholic, aOR = 2.5 Muslim each versus none/other). Implant versus non-LARC uptake was associated with having little or no education; meanwhile, having higher education was associated with IUD versus implant uptake.

**Conclusions:**

Fertility goal-based and couple-focused family planning counseling delivered by CHW, coupled with LARC training and support of nursing staff, substantially increased uptake of LARC methods.

## Plain English summary

Community health workers (CHW) play a central role in promoting healthcare in Rwanda. From August 2015 to September 2016, we trained CHW to promote long-acting reversible contraception (LARC) in communities in Kigali, the capital of Rwanda. CHW promoters counseled potential clients on fertility goal-based family planning to increase community awareness of LARC methods. They also engaged couples in this family planning counseling when possible and provided written referrals to clients expressing interest in LARC methods. We simultaneously trained government clinic nurses on LARC method insertions and removals. Over 7000 referrals were delivered by 184 CHWs from eight government clinics. Over 6000 family planning clinic visits resulted from these referrals. Almost all (95%) of these clinic visits resulted in LARC uptake (16% of women selected the copper intrauterine device uptake while 79% of women selected the hormonal implant). The monthly average for intrauterine device insertions doubled from 29 prior to service implementation to 61 after, and the monthly average for implant insertions increased from 109 to 309. Among women referred by a CHW, LARC uptake in the clinics was more likely if the referral had been issued to a couple (versus the woman alone) and if women had more children, desired fewer children, and had a religious affiliation. Overall, fertility goal-based and couple-focused family planning counseling delivered by CHWs, coupled with LARC training and support of nursing staff, increased uptake of LARC methods.

## Background

Rwanda, a small country in east central Africa, is characterized by high total fertility (roughly four children are born per woman) and high population density [1]. Unmet need for family planning affects almost 20% of women, and recent data show that almost half (47%) of Rwandan women would prefer not to have more children [2]. Improving family planning access is a top priority for the Rwandan Ministry of Health (MoH) [3–5]. In 2010, Rwanda endorsed delivery of injectable contraception for current users by community health workers (CHW) [6]. The central role of CHW was reiterated in the 2012 MoH family planning policy [7], in a consensus meeting about longer-acting injectable contraception in 2014 [8], and at the London Family Planning 2020 summit in 2017 [9].

For over 30 years, Projet San Francisco (PSF), based in Kigali, Rwanda and part of the Rwanda Zambia HIV Research Group (RZHRG), has led the implementation of couples’ voluntary HIV counseling and testing (CVCT), an evidence-based HIV prevention intervention [10]. Since 2009, Rwanda remains the only African country to have successfully implemented CVCT in antenatal clinics nationwide [11]. Drawing from experiences with CVCT, formative research conducted by PSF confirmed previous published work [12] that lack of familiarity with LARC methods, misconceptions about the safety and efficacy of LARC methods, and non-involvement of male partners were the primary client-side obstacles to scaling up LARC provision in Rwanda [13]. Provider bias has been reported in several studies as a persistent obstacle to provision of the full range of contraceptive services [14–16]. In Kigali, government clinic nurses who were not trained to insert LARC or did not have time to provide them were reluctant to promote them. To overcome these obstacles, since 2009 PSF has provided government clinics with training on long-acting reversible contraceptive (LARC) methods, including the copper intrauterine device (IUD) and contraceptive implant. These methods cannot be provided by CHW, but they offer many advantages including a low failure rate, less reliance on user adherence or consistent supply chains, and few adverse health effects [17]. Despite the clear applicability of LARC methods to the Rwandan context, in particular for couples wishing to limit or space pregnancies for at least two years, there has been historically low LARC access and uptake by Rwandan women [4, 5, 18], especially the IUD [1]. The IUD and implant comprised 0.7 and 4.7%, respectively, of the nationwide method mix among all women aged 15–49 per the 2014/2015 Rwandan Demographic Health Survey [19–21].

When CHW in Kigali assumed responsibility for distributing oral contraceptive pills (OCP) and providing injectable hormonal contraception in the community, PSF developed a training program to add information about LARC methods and family planning counseling, with a focus on couples family planning counseling ((C)FPC) techniques. In this manuscript, we present the results of a program that included fertility goal-based counseling and LARC promotion by CHW, with nurse training and logistical support in eight government clinics in Kigali, the capital city.

## Methods

### Ethics

This was programmatic research involving service delivery data unlinked to identifiers and exempt from Emory Institutional Review Board review.

### (C)FPC content and delivery by CHW

(C)FPC was developed from formative research with CHW, nurses, and CVCT clients, followed by a small pilot study in catchment areas for two Kigali clinics in 2014 [22]. Clients are educated about the full range of family planning options available, and fertility goal-based counseling identifies women and couples wishing to limit or delay conception for at least two years, the group for which LARC methods are most suitable. Standard FP counseling considers the client’s current desire or lack of desire for pregnancy. Fertility goal-based counseling goes beyond standard family planning counseling in that it considers long-, medium-, and short-term family planning goals. This facilitates discussion of birth spacing and other fertility planning considerations. Fertility goal-based counseling is a useful approach for determining when LARC methods may be appropriate, as opposed to short-acting methods or sterilization procedures (tubal ligation, vasectomy). Fertility goal-based counseling, when done as part of (C) FPC, also includes facilitated dialogue between partners about their fertility and family planning goals. This allows for discussion of misaligned or miscommunicated family planning goals between partners.

CHW affiliated with eight high-volume government clinics had previously been trained in family planning counseling, distribution of OCP, and administration of injectable hormonal contraception. For the (C)FPC program, CHW received additional training about LARC methods, the importance of engaging the man and woman together as a couple, and counseling based on fertility intentions. The CHW leveraged existing networks and rapport to approach the constituents who might be most interested in LARC. After (C)FPC, women and couples interested in receiving LARC were given written referrals to the clinic. Referrals did not expire. Referrals included the ID number of the distributing CHW, date of issue, and the PSF logo.

Because referrals did not expire, a clinic visit could occur in the same or a later month. The variation over time in referrals reflected the need to balance demand and supply: for example, many referrals were given in October and this led to a substantial rise in insertions. In order not to overwhelm the clinic nurses, the number of referrals given to CHW was reduced until February of 2016, and again reduced in March and increased in May 2016.

### LARC training for government clinic nurses

Prior to the launch of (C)FPC demand creation by CHW, family planning nurses from the same eight clinics received didactic and practical training in IUD and implant counseling, eligibility assessment, and insertion and removal. Didactic sessions were based on Rwanda MoH materials supplemented with additional information addressing misconceptions and gaps identified during formative work.

### Coordination of LARC community demand and government clinic supply

The (C)FPC program was launched in August 2015 and ended in September 2016. Prior to launch, adequate supplies and equipment for the clinics were ensured through negotiation with government commodity providers. Regular meetings were scheduled between nurses and CHW to discuss logistics and enhance collaboration. As CHW increased the client-side demand for LARC, the clinic-side capacity for LARC provision was scaled up through supplemental staffing and material support. The number of referrals distributed by CHW was carefully controlled, in order not to overwhelm clinic capacity while maintaining steady and strong attendance at family planning clinics. As clinic nurses became more comfortable and efficient with LARC service provision, the number of CHW trained in (C)FPC and the number of referrals each could distribute was increased accordingly.

Building on the existing performance-based financing (PBF) pay system provided by government as an incentive for good performance, clinics were reimbursed $0.63USD for each insertion [23, 24]. As is the norm for CHW, their remuneration was based on performance, and they were paid a modest sum for each woman or couple presenting to the clinic with their referral (regardless of whether LARC was requested).

### Data recording and extraction

During (C)FPC implementation, we recorded the number of CHW referrals issued and resulting family planning clinic visits and recorded the contraceptive method type selected in logbooks. The number of referrals distributed by CHW was recorded as were their referral slips returned by clients to clinic. To facilitate service provision at the clinic, basic demographic data were recorded including woman’s educational level, religion, marital status, number of living and desired children, current contraceptive method, and whether the woman alone (FPC) or the couple together ((C)FPC) received the CHW referral. Pre- and post-implementation data were collected by CHW and/or clinic staff in hard copy logbooks, and later extracted by PSF staff for data entry in Microsoft Access.

### LARC method uptake before and after (C)FPC implementation

To assess the impact of the (C)FPC implementation, we used clinic records to extract data on monthly IUD and implant (Jadelle or Implanon) insertions in the year prior to our implementation, August 2014 to August 2015. One clinic did not have comparable data available, and was excluded from this analysis only (all other analyses consider all 8 clinics). Pre- versus post- comparisons (overall, by method selected, and by health clinic) were made using two-tailed paired t-tests in SAS v9.4 (Cary, NC). The threshold for statistical significance was *p* < 0.05 for all tests.

### Client demographics and correlates of LARC uptake

Demographic data are described using counts and percentages or means and standard deviations (SDs) for categorical and continuous variables, respectively. Women were categorized as “LARC uptakers” (adopted a new LARC method, or retained/replaced a current LARC method), or as “non-LARC uptakers” (did not adopt a new LARC method, or switched from LARC to a non-LARC method). Chi-square (of Fisher’s exact) tests and t-tests, as appropriate, evaluated differences in women who selected any LARC, IUD, or implant (each versus non-LARC uptake) as well as those who selected the IUD versus the implant. Demographics associated with LARC, IUD, or implant uptake (each versus non-LARC uptake) or the IUD versus the implant in bivariate analyses were evaluated in multivariate logistic regression models using SAS v9.4 (Cary, NC) after assessing for variable collinearity. Adjusted odds ratios (aORs) and 95% confidence intervals (CIs) are reported.

## Results

### CHW referrals and LARC insertions during (C)FPC implementation

**Fig. 1:**
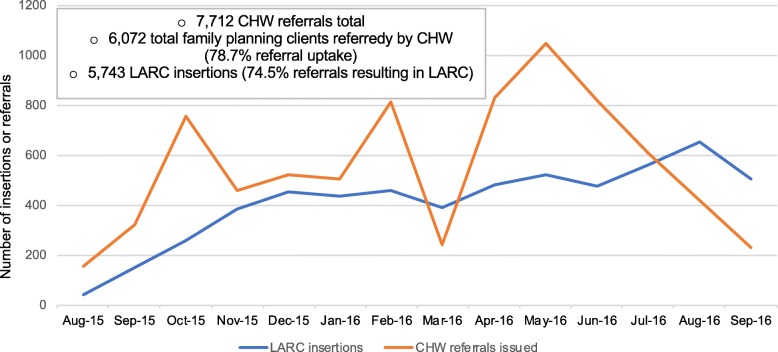
Monthly number of LARC insertions and CHW-issued referrals (*n* = 8 clinics). LARC: Long-acting reversible contraceptive. CHW: Community Health Worker. Since referrals could lead to clients arriving for family planning services in the following months, referrals are not matched to uptake date

184 CHW issued 7712 referrals, resulting in 6072 family planning visits at the eight government clinics, an overall referral uptake of 78.7%. As shown in Fig. 1 and described in the methods section, the number of CHW referrals per month varied over time to reflect the need to balance demand and supply. The number of LARC insertions increased over time, with 39 insertions in the first month and peaking at 649 in the penultimate month of the program (August 2016). The average time between referral and LARC uptake was 5.5 days.

Among the 6072 family planning clients, over one-half (57%) were not contracepting or were relying on condoms or traditional methods to prevent pregnancy. After (C)FPC, 94.6% selected a LARC method (79.1% implant, 15.5% IUD), while 5.2% selected no new method, 0.2% selected the injectable, and < 0.1% selected OCP (data not shown). For clients receiving a LARC method, the interval between the initial (C)FPC session with the CHW and the insertion of the LARC ranged from 0 to 63 days.

### LARC uptake pre- and post-(C)FPC implementation

**Fig. 2:**
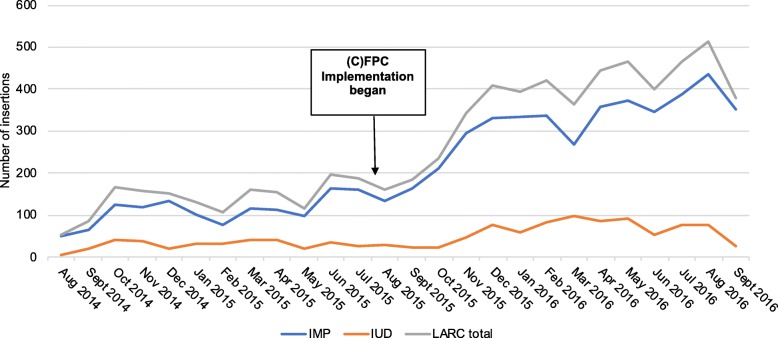
Comparison of monthly number of LARC insertions pre- and post-(C)FPC implementation (*n* = 7 clinics*). LARC: Long-acting reversible contraceptive. (C)FPC: (Couples) Family Planning Counseling. IMP: Contraceptive implant. IUD: Intra-uterine device. Change in monthly insertions pre-post (C)FPC implementation (paired t test): p-value overall < 0.0001, p-value implant < 0.0001, p-value IUD < 0.0001. Change in monthly insertions pre-post (C)FPC implementation, by clinic (paired t test): *p* < 0.001 for overall: all clinics except one clinic, *p* < 0.001 for implant: all clinics except one clinic, *p* < 0.001 for IUD: all clinics except five clinics. *Pre-implementation data not recorded consistently for one of the eight clinics, which is not included in this figure

Seven clinics had comparable pre-implementation data for this analysis. Taken in aggregate, there was a meaningful and statistically significant increase in overall LARC uptake pre- versus post-implementation (*p* < 0.0001) (Fig. 2). The monthly average for IUD insertions increased from 29 (SD = 11.5) to 61 (SD = 27.4) (*p* < 0.0001), and the monthly average for implant insertions increased from 109 (SD = 34.5) to 309 (SD = 86.8) (*p* < 0.0001). By clinic, six clinics significantly increased monthly implant uptake, while only two clinics significant increased monthly IUD uptake (all *p* < 0.001).

### (C)FPC client demographics and correlates of LARC uptake (Table 1)

Comparisons are statistically significant unless otherwise specified. Of the 6072 clients presenting to the family planning clinic with a referral from a CHW, 329 (5.4%) were non-LARC uptakers, and 5743 (94.6%), were LARC uptakers. Implant was the most popular LARC method selected, with 4803 (83.6%) selecting that method compared to 940 women (16.4%) selecting IUD. Of those receiving a LARC method, 37% (41% of IUD and 36% of implant uptakers) had received (C)FPC with their partners. In comparison, only 22% of non-LARC uptakers had received counseling with their partners.

**Table 1:** Demographics by LARC method uptake among (C)FPC clients (*n* = 8 clinics)

	Non-LARC uptakers (*N* = 329)		LARC uptakers (*N* = 5743)		*p*-value^a^	IUD uptakers (*N* = 940)		*p*-value^b^	Implant uptakers (*N* = 4803)		*p*-value^c^	*p*-value^d^
	N	%	N	%		N	%		N	%		
Age (mean, SD)	28.4	5.3	28.4	5.9	> 0.999	29.0	5.3	0.078	28.3	6.0	0.743	<.001
Referral issued to					<.0001			<.0001			<.0001	0.005
Woman alone	257	78%	3589	63%		550	59%		3039	64%		
Couple	72	22%	2117	37%		385	41%		1732	36%		
Educational Level					0.235			0.001			0.005	<.0001
None	9	3%	263	5%		12	1%		251	5%		
Primary	176	60%	3622	63%		477	51%		3145	66%		
Secondary	92	32%	1633	29%		367	39%		1266	27%		
Higher Degree	14	5%	194	3%		80	9%		114	2%		
Religion					<.0001			0.089			<.0001	<.0001
Protestant	141	49%	2882	50%		405	43%		2477	52%		
Catholic	114	39%	2475	43%		440	47%		2035	43%		
Muslim	7	2%	129	2%		25	3%		104	2%		
None/Other	28	10%	227	4%		65	7%		162	3%		
Marital status					0.233			<.0001			0.095	<.0001
Legally married	148	51%	2721	48%		565	60%		2156	45%		
Cohabiting/common law-union	113	39%	2505	44%		336	36%		2169	45%		
Single, widowed, separated	29	10%	485	8%		35	4%		450	9%		
Number of children living (mean, SD)	2.0	1.2	2.2	1.3	0.004	2.2	1.2	0.010	2.2	1.3	0.004	1.000
Number of additional children desired (mean, SD)	2.5	1.3	2.2	1.5	<.0001	2.0	1.4	<.0001	2.3	1.5	0.008	<.0001
Current contraceptive method ^e^					<.0001			0.005			<.0001	<.0001
Condoms/none/traditional ^f^	155	47%	3308	58%		522	56%		2786	58%		
Injectable	107	33%	1629	28%		226	24%		1403	29%		
OCPs	66	20%	564	10%		173	18%		391	8%		
Implant	1	0%	226	4%		14	1%		212	4%		
IUD	0	0%	15	0%		5	1%		10	0%		
Collapsed categories current contraceptive					<.0001			0.001			<.0001	<.0001
Condoms/none/traditional	155	47%	3308	58%		522	56%		2786	58%		
Injectable/OCPs	173	53%	2193	38%		399	42%		1794	37%		
LARC	1	0%	241	4%		19	2%		222	5%		

Counts may not sum to total due to missingness

*LARC* Long-acting reversible contraceptive. *(C)FPC* (Couples) Family Planning Counseling, *IMP* Contraceptive implant, *IUD* Intra-uterine device, *SD* standard deviation

*P*-values from Chi-square (or Fisher’s exact) tests for categorical variables or t-tests for continuous variables are two-sided

^a^LARC uptakers vs. non-LARC uptakers

^b^IUD uptakers vs. non-LARC uptakers

^c^Implant uptakers vs. non-LARC uptakers

^d^Implant vs IUD

^e^Current reported contraceptive use on the day women came to the clinic with a community health worker referral

^f^Most (> 95%) of these method users are no method users with occasional condom use; traditional method users are combined given similar typical use-failure rates

Women were 28 years of age on average. Most women had a primary (63%) or secondary (29%) educational level, with those receiving an IUD more likely to have secondary or higher education (48% vs 29% of implant uptakers and 37% of non-LARC uptakers). Most women were either Protestant (50%) or Catholic (43%), with a higher proportion of non-Christians in the non-LARC group (12% vs 6% in the LARC group) and the highest proportion of Catholics among the IUD uptakers (47% vs 43% of implant uptakers and 39% of the non-LARC uptakers). Most were either married (48%) or in a traditional or common law cohabiting union (44%), with the IUD group having the highest proportion of legal marriages (60% vs 45% of implant and 51% of non-LARC groups). The non-LARC group had fewer living children (average 2.0) than the LARC group (average 2.2). Likewise, the non-LARC group desired a greater number of additional children (average 2.5) than the LARC group (average 2.2). Within the LARC group, IUD uptakers desired fewer children than the Implant users (average 2.0 vs 2.3).

Current reported contraceptive use on the day women came to the clinic with a CHW referral included injectable (29%), OCP (10%), implant (4%), IUD (< 1%), and none/condoms only/traditional methods (57%). Non-LARC uptakers were more likely to be using OCP or injectable hormonal contraception, while women requesting an IUD or implant were more likely to be using only condoms, traditional methods, or no contraception. Among *n* = 242 women using LARC methods at the time of their CFPC visit (Table 1), all but one selected a LARC method (*n* = 212/227 previous implant users had implants reinserted, 14/227 previous implant users selected the IUD, 5/15 previous IUD users had IUDs reinserted, and 10/15 IUD users selected the implant).

### Multivariate analyses of predictors of IUD and implant uptake (Table 2)

Comparisons included in the text are statistically significant unless specified. In adjusted analyses, LARC uptake versus non-LARC uptake was associated with the CHW referral being issued to the couple (versus the woman alone; aOR = 2.57); the woman’s religion being Protestant (aOR = 2.89), Catholic (aOR = 3.05), or Muslim (aOR = 2.50) versus other or no religion; having more living children (aOR = 1.26 per child); desiring fewer children (aOR = 0.84 per child); already using a LARC (aOR = 7.86) or injectable/OCP (aOR = 0.45) method versus condom use.

**Table 2:** Adjusted factors associated with LARC method uptake among (C)FPC clients (n = 8 clinics)

	Any LARC uptake versus non-LARC uptake				IUD uptake versus non-LARC uptake				Implant uptake versus non-LARC uptake				IUD uptake versus implant uptake			
	aOR	95% CI		*p*-value	aOR	95% CI		*p*-value	aOR	95% CI		*p*-value	aOR	95% CI		*p*-value
Referral issued to																
Woman alone	ref				ref				ref				ref			
Couple	2.57	1.91	3.45	<.0001	2.92	2.10	4.06	<.0001	2.55	1.89	3.44	<.0001	1.18	1.01	1.37	0.035
Educational Level																
None/Primary					ref				ref				ref			
Secondary					1.37	1.01	1.87	0.044	0.76	0.58	0.99	0.044	1.98	1.70	2.32	<.0001
Higher Degree					1.65	0.88	3.11	0.119	0.41	0.23	0.74	0.003	3.86	2.82	5.30	<.0001
Religion																
Protestant	2.89	1.86	4.48	<.0001					3.12	1.98	4.91	<.0001	0.46	0.33	0.64	<.0001
Catholic	3.05	1.95	4.77	<.0001					3.23	2.04	5.23	<.0001	0.60	0.43	0.83	0.001
Muslim	2.50	1.05	5.97	0.038					2.70	1.12	6.55	0.027	0.63	0.37	1.09	0.100
None/Other	ref								ref				ref			
Marital status																
Legally married					2.73	1.54	4.84	0.001					3.02	2.10	4.35	<.0001
Cohabiting					1.90	1.07	3.37	0.033					1.92	1.32	2.77	0.001
Single, widowed, separated					ref								ref			
No. of children living (per child increase)	1.26	1.14	1.41	<.0001	1.21	1.06	1.37	0.004	1.23	1.11	1.38	<.001				
No. of additional children desired (per child increase)	0.84	0.77	0.92	<.0001	0.72	0.64	0.80	<.0001	0.86	0.79	0.94	0.001	0.87	0.83	0.92	<.0001
Current contraceptive ^a^																
Condoms/none/traditional^b^	ref				ref				ref				ref			
Injectable/OCPs	0.45	0.36	0.58	<.0001	0.57	0.42	0.76	<.001	0.42	0.33	0.55	<.0001	1.29	1.11	1.50	0.001
LARC	7.86	1.09	56.68	0.041	3.18	0.41	24.64	0.268	8.20	1.14	59.21	0.037	0.52	0.32	0.85	0.009

P-values from logistic regression models are two-sided

*LARC* Long-acting reversible contraceptive, *(C)FPC* (Couples) Family Planning Counseling, *IMP* Contraceptive implant, *IUD* Intra-uterine device, *aOR* adjusted odds ratio, *CI* confidence interval

^a^Current reported contraceptive use on the day women came to the clinic with a community health worker referral

^b^Most (> 95%) of these method users are no method users with occasional condom use; traditional method users are combined given similar typical use-failure rates

When IUD uptake alone was compared with non-LARC uptake, the predictors above remained significant except for current LARC use and religious affiliation. In addition, having a secondary education versus none/primary education (aOR = 1.37) and being married (aOR = 2.73) or cohabiting (aOR = 1.90) (both versus being single, widowed, or separated) were associated with IUD uptake.

Corresponding comparisons of implant users with non-LARC uptakers showed the same predictors as the model for LARC vs non-LARC uptake as a whole, with the exception of lower likelihood of implant uptake with secondary (aOR = 0.76) or higher education (aOR = 0.41) versus none/primary education.

IUD versus implant uptake was associated with CHW referral being issued to the couple (aOR = 1.18) (versus the woman alone); secondary (aOR = 1.98) or higher education (aOR = 3.86) (versus none/primary education); and being in a legal (aOR = 3.03), traditional, or common law cohabiting union (aOR = 1.92) (versus being single, widowed, or separated). Women who selected IUD were less likely to be Protestant (aOR = 0.46) or Catholic (aOR = 0.60), and desired fewer future children (aOR = 0.87) than women choosing implant, and were less likely to report prior LARC use (aOR = 0.52) than women choosing implant.

## Discussion

Fertility goal-based family planning counseling delivered by CHW in their community, and conducted in tandem with LARC nurse training and logistical support in government clinics, was associated with a substantial and rapid increase in LARC uptake. This was particularly true when both partners in the couple received the counseling. Partner participation was also associated with higher likelihood of choosing the IUD over the implant, though overall the preference for implants was maintained. LARC uptake was also associated with demographic characteristics which may be used to refine promotional messages and counseling content. In particular, IUD uptake was associated with being legally married while implant use was associated with women having little or no education.

Our services included many components that have been previously assessed in other programs and countries. While the impact of the particular combination is likely more than a sum of its parts, we include here discussion of task shifting to CHW, targeted fertility goal-based messaging, inclusion of men, and comparisons of clients who chose the implant versus IUD.

The timing of these services in Kigali was deliberate: PSF’s previous efforts had concentrated on training Kigali government clinic nurses to promote and provide LARC in the clinics and did not include community-based promotion. That strategy worked well when OCP and injectable clients returned to the clinic quarterly for their injection or re-supply. They could hear about LARC on multiple occasions and choose to ‘upgrade’ at any time. When follow-up OCP and injectable provision was task shifted to CHW [25], the clinic audience for LARC promotions was limited to women initiating a method who would hear the message only once. Having LARC messages repeated by CHW addresses a key component of the WHO Strategic Communications Framework for effective communications [26]. Rwandan CHW are assigned a specific set of households, are familiar with the individual health needs of adults and infants including family size, OCP and injectable use, and the need for child spacing. This allowed them to focus their promotions on couples and women who were most likely to be receptive to fertility goal-based counseling, a strategy that succeeded as evidenced by the high uptake (79%) of CHW referrals. A similar voucher system with CHW workers was also successful in increasing uptake in Uganda [27]. In order not to overwhelm the clinic nurses, the number of referrals given to CHW was reduced through March 2016 and increased in May 2016. This can be seen in Fig. 1, where *n* = 244 referrals were issued in March while *n* = 1044 were issued in May. These decisions were made based on reports from clinic nurses regarding workload. Though we did not explore it here, a more systematic method for determining the number of referrals provided to CHW could be useful.

Male partner participation has long been encouraged in family planning programs. In Ethiopia, awareness and information was necessary to foster partners’ payment for transport and contraceptive services [28]. In Malawi [29] and Ghana [30], family planning education provided to the couple together was more effective than addressing the woman alone. In our study, when CHW counseled both partners together (versus the woman alone), women were almost three times more likely to choose a LARC method. This was not surprising given our previous research that referrals to the couple together also led to increased uptake of CVCT [31, 32]. Distribution of CHW referrals to the couple together was also associated with IUD versus implant uptake, perhaps because increasing IUD education among both partners is critical given relatively low baseline knowledge and use of that method [13, 33–35]. If a male partner was not available, CHW counseled women alone and the majority of LARC clients were in this group. This suggests that programs may do well to emphasize couples when possible but given practical constraints they should not limit themselves only to couples.

Several demographic characteristics were associated with LARC uptake in Kigali. Interestingly, being of any religion, including Catholic, was associated with implant versus non-LARC uptake and was also associated with implant versus IUD uptake. This is surprising given traditional Catholic viewpoints on contraception, but may reflect the tempering of the church’s mandate on contraception in Rwanda, as well as a shift to prioritize politics and Ministry of Health recommendations over religion [36]. The fact that common preconceptions about Catholics did not hold is encouraging for future family planning programs, especially in countries such as Rwanda where a large proportion of the population is Catholic.

Another unexpected finding was the association of implant choice with little or no education, and the comparatively higher education among IUD compared with implant clients. Almost half of women who selected the IUD had a secondary education or higher, while 71% of implant users had no or only primary education. Most studies have found that higher education is associated with LARC uptake [37, 38], but these studies have not compared demographic differences in IUD versus implant uptake. It is possible that less educated women are still exposed to and aware of the hormonal implant simply because it is a more well-known method, but that only women with higher education are receiving information about the IUD. This reinforces the finding above that to improve IUD uptake, concerted educational efforts focusing on the IUD need to be incorporated into programs as has been successfully done in other studies [35].

Several demographic characteristics associated with LARC uptake were consistent with the literature. Having more children and wanting fewer additional children predicted LARC uptake. There is ongoing need to promote LARC methods among clients with young infants as LARC is ideal for safely spacing births to improve maternal and infant health outcomes [39]. Current LARC users were more likely to request a replacement of their LARC method (primarily expiring implants) or to switch to the other LARC method than they were to discontinue LARC use. Finally, women who selected the IUD were more likely to be legally married or cohabiting versus women who selected non-LARC methods or the implant. Again, as prior studies have not explored in-depth the differences between women who select the IUD versus implant, this finding warrants further research.

A preference for implants compared with the IUD has been reported in several settings. During a large scale-up of LARC provision in 15 sub-Saharan African countries between 2008 and 2012, Marie Stopes International researchers noted a much higher uptake of implants versus IUDs [40]. A study of LARC scale-up in Chad and the Democratic Republic of Congo in 2011 also found very high demand for implants which comprised most of the method mix [35]. The implant is a more well-known and commonly used method versus the IUD in much of sub-Saharan Africa including Rwanda [13, 33–35]. and providers may be less comfortable inserting IUDs as more skill is required [41–43]. Client preference for implants should be supported after clients receive complete, unbiased information about all method options. If providers prefer to counsel on and insert implants relative to IUDs, as this may interfere with their ability to provide full, unbiased family planning method information to all clients. To increase IUD demand, concerted promotional efforts must be made to overcome client misconceptions [44, 45], while provider bias in favor of implants must be overcome with training and mentorship [46].

Integration of family planning and HIV services has broad support [64–66] and high-impact to decrease unmet need for contraception, unplanned pregnancy, and perinatal HIV transmission [67]. Rwanda’s HIV epidemic is generalized with a national prevalence of 3% (6.2% urban, 2.2% rural; 3.6% women, 2.2% men nationally; 8.0% women, 4.4% men in Kigali) [1]. (C)FPC and CVCT could be readily integrated. PSF has conducted research on the integration of family planning and HIV services; the impact of hormonal contraception and pregnancy on HIV incidence and disease progression; and the associations between pregnancy, genital inflammation and ulceration, and HIV transmission [47–63], and (C)FPC and CVCT share PSF’s signature couples-based model. The role of CHW in the nationwide expansion of CVCT was pivotal, and the two messages can be readily integrated to provide cohesive HIV and unplanned pregnancy prevention [47]. Since heterosexual couples form the largest risk group for both HIV and unplanned pregnancy in Rwanda, integrating HIV and family planning counseling for couples is a priority.

Some limitations warrant discussion. Data about the reasons for non-LARC uptake (non-eligibility for LARC versus preference for another contraceptive method after discussion with a nurse) among the 5.4% of non-LARC clients would have been informative. Misclassification, possibly differential by the outcomes of interest, is possible with self-reported client data which could bias our results in an unknown direction. The selection of clinics was not a representative and generalizable sample, but rather a prioritization of clinics that might be appropriate for future expansion of (C)FPC. Though infrequent, demographic client data could be missing if data was not recorded in government clinic logbooks, which could occur for a variety of reasons (e.g., provider forgot to ask, client refused). Finally, though we use data from the same intervention clinics as historical controls, we did not collect data from other facilities as contemporaneous controls. Though the pre- versus post- evaluation design is susceptible to secular trends, the absence of major shifts in other influencing factors, such as level of government support for LARC, cost of LARC insertion, and other LARC capacity building projects taking place in target areas during this time period allows us to reasonably assume that changes in LARC uptake are largely attributable to (C)FPC programming.

## Conclusions

Overall, the (C)FPC evaluation yielded promising results. A community-based promotional model where CHW promote LARC through fertility goal-based counseling was an effective service that rapidly increased LARC uptake in Rwanda. The success of these implementation efforts relied on a careful balance between the scaling-up of LARC provision capacity with the scaling-up of LARC promotion in the community by CHW, with ongoing monitoring and evaluation efforts.

## Abbreviations

(C)FPC: Couples’family planning counseling
aOR: Adjusted odds ratio
CFPC: Couples’ family planning counseling
CHW: Community health worker
CVCT: Couples’ voluntary HIV counseling and testing
IUD: Intrauterine device
LARC: Long-acting reversible contraception
MoH: Ministry of Health
OCP: Oral contraceptive pills
PSF: Projet San Francisco
RZHRG: Rwanda Zambia HIV Research Group

## References

[1] National Institute of Statistics of Rwanda (NISR) [Rwanda], Ministry of Health (MOH) [Rwanda], ICF International. Rwanda Demographic and Health Survey Rockville, Maryland, USA: NISR, MOH, and ICF International; 2015 [Available from: https://dhsprogram.com/pubs/pdf/FR316/FR316.pdf.

[2] Rwanda Demographic and Health Survey. Trends in Reproductive Behavior in Rwanda: Further Analysis of the 2014–15 Rwanda. Demographic and Health Survey Rockville, Maryland: ICF; 2014–15 [Available from: https://dhsprogram.com/pubs/pdf/FA107/FA107.pdf.

[3] Republic of Rwanda Ministry of Health. Family planning strategic plan 2012–2016: Rwandan Ministry of Health; 2012 [Available from: http://www.moh.gov.rw/fileadmin/templates/Docs/Rwanda-Family-Planning-Strategic-2012-2013.pdf.

[4] Ayad M, Hong R (2009). Further Analysis of the Rwanda Demographic and Health Surveys, 2000–2007/08: Levels and Trends of Contraceptive Prevalence and Estimate of Unmet Need for Family Planning in Rwanda Calverton, Maryland.

[5] United Nations (2015). Department of economic and social affairs, population division. Trends in contraceptive use worldwide 2015.

[6] USAID (2010). Community-Based distribution of injectable contraceptives in Rwanda: an intervention to reverse rural disadvantage health Policv InitiativeFutures Group.

[7] Rwandan Ministry of Health (2012). Family Planning Policy Rwanda.

[8] McKenna K, Arcara J, Rademacher KH, Mackenzie C, Ngabo F, Munyambanza E (2014). Policy and programmatic considerations for introducing a longer-acting injectable contraceptive: perspectives of stakeholders from Kenya and Rwanda. Glob Health Sci Pract.

[9] Rwandan Ministry of Health (2017). Family Planning 2020 Commitment London.

[10] Dunkle KL, Greenberg L, Lanterman A, Stephenson R, Allen S (2008). Source of new infections in generalised HIV epidemics – authors' reply. Lancet.

[11] Karita E, Nsanzimana S, Ndagije F, Wall KM, Mukamuyango J, Mugwaneza P (2016). Implementation and operational research: evolution of Couples' voluntary counseling and testing for HIV in Rwanda: from research to public health practice. J Acquir Immune Defic Syndr (1999).

[12] Brunie A, Tolley EE, Ngabo F, Wesson J, Chen M (2013). Getting to 70%: barriers to modern contraceptive use for women in Rwanda. Int J Gynaecol Obstet.

[13] Grabbe K, Stephenson R, Vwalika B, Ahmed Y, Vwalika C, Chomba E (2009). Knowledge, use, and concerns about contraceptive methods among sero-discordant couples in Rwanda and Zambia. J Women's Health (Larchmt).

[14] Mayhew SH, Colombini M, Kimani JK, Tomlin K, Warren CE, Integra I (2017). Fertility intentions and contraceptive practices among clinic-users living with HIV in Kenya: a mixed methods study. BMC Public Health.

[15] Sullivan TM, Bertrand JT, Rice J, Shelton JD (2006). Skewed contraceptive method mix: why it happens, why it matters. J Biosoc Sci.

[16] Schwandt HM, Speizer IS, Corroon M (2017). Contraceptive service provider imposed restrictions to contraceptive access in urban Nigeria. BMC Health Serv Res.

[17] World Health Organization (2018). Contraception Geneva, Switzerland: WHO Press.

[18] Rwandan Ministry of Health (2017). Rwanda FP2020 Core Indicator summary sheet.

[19] National Institute of Statistics of Rwanda (NISR), Ministry Of Health, ICF International. 2014–15 Rwanda Demographic Health Survey Key Findings Rockville, Maryland, USA: Rockville, Maryland, USA; 2015 [Available from: http://www.dhsprogram.com/pubs/pdf/SR229/SR229.pdf.

[20] Rwandan Ministry of Health. Rwanda demographic and health survey 2014–15 final report: National Institute of Statistics of Rwanda (NISR), Ministry of Health Rwanda, The DHS Program, ICP International; 2015 [Available from: https://dhsprogram.com/pubs/pdf/FR316/FR316.pdf.

[21] National Institute of Statistics of Rwanda (NISR), Rwandan Ministry of Health, ICF International. Rwanda Demographic and Health Survey. Rockville, Maryland, USA: NISR, MOH, and ICF International; 2015.

[22] Ingabire R, Karita E, Ahmed N, Bayingana R, Nyombayire JM, Sinabamenye R (2014). Capacity Strengthening and Training of Government Nurses on Long-acting Reversible Contraceptive (LARC) Methods in Kigali, Rwanda.

[23] Bucagu M, Kagubare JM, Basinga P, Ngabo F, Timmons BK, Lee AC (2012). Impact of health systems strengthening on coverage of maternal health services in Rwanda, 2000-2010: a systematic review. Reprod Health Matters.

[24] Rusa L, Ngirabega Jde D, Janssen W, Van Bastelaere S, Porignon D, Vandenbulcke W (2009). Performance-based financing for better quality of services in Rwandan health centres: 3-year experience. Tropical Med Int Health.

[25] Chin-Quee D, Mugeni C, Nkunda D, Uwizeye MR, Stockton LL, Wesson J (2016). Balancing workload, motivation and job satisfaction in Rwanda: assessing the effect of adding family planning service provision to community health worker duties. Reprod Health.

[26] World Health Organization (2017). Strategic communications framework.

[27] Bellows B, Mackay A, Dingle A, Tuyiragize R, Nnyombi W, Dasgupta A (2017). Increasing contraceptive access for hard-to-reach populations with vouchers and social franchising in Uganda. Glob Health Sci Pract..

[28] Balogun O, Adeniran A, Fawole A, Adesina K, Aboyeji A, Adeniran P (2016). Effect of male Partner's support on spousal modern contraception in a low resource setting. Ethiop J Health Sci.

[29] Bryant AG, Hamela G, Gotter A, Stuart GS, Kamanga G (2015). Reasons for intrauterine device use, discontinuation and non-use in Malawi: a qualitative study of women and their partners. Afr J Reprod Health.

[30] Cox CM, Hindin MJ, Otupiri E, Larsen-Reindorf R (2013). Understanding couples' relationship quality and contraceptive use in Kumasi, Ghana. Int Perspect Sex Reprod Health.

[31] Wall K, Karita E, Nizam A, Bekan B, Sardar G, Casanova D (2012). Influence network effectiveness in promoting couples' HIV voluntary counseling and testing in Kigali, Rwanda. Aids..

[32] Wall Kristin M, Kilembe William, Nizam Azhar, Vwalika Cheswa, Kautzman Michelle, Chomba Elwyn, Tichacek Amanda, Sardar Gurkiran, Casanova Deborah, Henderson Faith, Mulenga Joseph, Kleinbaum David, Allen Susan (2012). Promotion of couples’ voluntary HIV counselling and testing in Lusaka, Zambia by influence network leaders and agents. BMJ Open.

[33] Tibaijuka L, Odongo R, Welikhe E, Mukisa W, Kugonza L, Busingye I (2017). Factors influencing use of long-acting versus short-acting contraceptive methods among reproductive-age women in a resource-limited setting. BMC Womens Health.

[34] Gutin S, Mlobeli R, Moss M, Buga G, Morroni C. Survey of knowledge, attitudes and practices surrounding the intrauterine device in south Africa2011. p. 145–50. 10.1016/j.contraception.2010.07.009 21237340

[35] Rattan J, Noznesky E, Curry DW, Galavotti C, Hwang S, Rodriguez M (2016). Rapid contraceptive uptake and changing method mix with high use of long-acting reversible contraceptives in crisis-affected populations in Chad and the Democratic Republic of the Congo. Glob Health.

[36] Wadhams N (2010). Progress in Rwanda's drive to slow population growth. Lancet.

[37] Blumenthal PD, Shah NM, Jain K, Saunders A, Clemente C, Lucas B (2013). Revitalizing long-acting reversible contraceptives in settings with high unmet need: a multicountry experience matching demand creation and service delivery. Contraception..

[38] Anyanwu M, Alida BWN (2017). Uptake of long-acting reversible contraceptive devices in Western region of the Gambia. Afr Health Sci.

[39] Wendt A, Gibbs CM, Peters S, Hogue CJ (2012). Impact of increasing inter-pregnancy interval on maternal and infant health. Paediatr Perinat Epidemiol.

[40] Duvall S, Thurston S, Weinberger M, Nuccio O, Fuchs-Montgomery N (2014). Scaling up delivery of contraceptive implants in sub-Saharan Africa: operational experiences of Marie stopes international. Glob Health Sci Pract..

[41] Robinson N, Moshabela M, Owusu-Ansah L, Kapungu C, Geller S (2016). Barriers to intrauterine device uptake in a rural setting in Ghana. Health Care Women Int.

[42] Greene E, Stanback J (2012). Old barriers need not apply: opening doors for new contraceptives in the developing world. Contraception..

[43] Osei I, Birungi H, Addico G, Askew I, Gyapong JO (2005). What happened to the IUD in Ghana?. Afr J Reprod Health.

[44] Twesigye R, Buyungo P, Kaula H, Buwembo D (2016). Ugandan Women's view of the IUD: generally favorable but many have misperceptions about health risks. Glob Health Sci Pract..

[45] Tilahun Y, Mehta S, Zerihun H, Lew C, Brooks MI, Nigatu T (2016). Expanding access to the intrauterine device in public health facilities in Ethiopia: a mixed-methods study. Glob Health Sci Pract..

[46] Goldstuck ND (2014). Reducing barriers to the use of the intrauterine contraceptive device as a long acting reversible contraceptive. Afr J Reprod Health.

[47] Khu NH, Vwalika B, Karita E, Kilembe W, Bayingana RA, Sitrin D (2013). Fertility goal-based counseling increases contraceptive implant and IUD use in HIV-discordant couples in Rwanda and Zambia. Contraception..

[48] Joseph Davey DL, Wall KM, Kilembe W, Khu NH, Brill I, Vwalika B (2018). Difficult decisions: evaluating individual and couple-level fertility intentions and HIV acquisition among HIV serodiscordant couples in Zambia. PLoS One.

[49] Haddad LB, Wall KM, Kilembe W, Vwalika B, Khu NH, Brill I (2018). Bacterial vaginosis modifies the association between hormonal contraception and HIV acquisition. AIDS..

[50] Wu KY, Oppert M, Wall KM, Inambao M, Simpungwe MK, Ahmed N, et al. Couples' voluntary HIV counseling and testing provider training evaluation, Zambia. Health Promot Int. 2017. 10.1093/heapro/daw108 PMC6144772 28119330

[51] Wall KM, Rida W, Haddad LB, Kamali A, Karita E, Lakhi S (2017). Pregnancy and HIV disease progression in an early infection cohort from five African countries. Epidemiology..

[52] Wall KM, Kilembe W, Vwalika B, Haddad LB, Khu NH, Brill I (2017). Optimizing prevention of HIV and unplanned pregnancy in discordant African couples. J Women's Health (Larchmt).

[53] Wall KM, Kilembe W, Vwalika B, Ravindhran P, Khu NH, Brill I (2016). Hormonal contraceptive use among HIV-positive women and HIV transmission risk to male partners, Zambia, 1994-2012. J Infect Dis.

[54] Wall KM, Kilembe W, Haddad L, Vwalika B, Lakhi S, Khu NH (2016). Hormonal contraception, pregnancy, breastfeeding, and risk of HIV disease progression among Zambian women. J Acquir Immune Defic Syndr (1999)..

[55] Wall KM, Vwalika B, Haddad L, Khu NH, Vwalika C, Kilembe W (2013). Impact of long-term contraceptive promotion on incident pregnancy: a randomized controlled trial among HIV-positive couples in Lusaka, Zambia. J Acquir Immune Defic Syndr (1999).

[56] Wall KM, Haddad L, Vwalika B, Htee Khu N, Brill I, Kilembe W (2013). Unintended pregnancy among HIV positive couples receiving integrated HIV counseling, testing, and family planning services in Zambia. PLoS One.

[57] Conkling M, Shutes EL, Karita E, Chomba E, Tichacek A, Sinkala M (2010). Couples' voluntary counselling and testing and nevirapine use in antenatal clinics in two African capitals: a prospective cohort study. J Int AIDS Soc.

[58] Hageman KM, Karita E, Kayitenkore K, Bayingana R, van der Straten A, Stephenson R (2009). What the better half is thinking: a comparison of men's and women's responses and agreement between spouses regarding reported sexual and reproductive behaviors in Rwanda. Psychol Res Behav Manag.

[59] Dunkle KL, Stephenson R, Karita E, Chomba E, Kayitenkore K, Vwalika C (2008). New heterosexually transmitted HIV infections in married or cohabiting couples in urban Zambia and Rwanda: an analysis of survey and clinical data. Lancet (London, England).

[60] Allen S, Stephenson R, Weiss H, Karita E, Priddy F, Fuller L (2007). Pregnancy, hormonal contraceptive use, and HIV-related death in Rwanda. J Women's Health (Larchmt).

[61] Allen S, Karita E, Chomba E, Roth DL, Telfair J, Zulu I (2007). Promotion of couples' voluntary counselling and testing for HIV through influential networks in two African capital cities. BMC Public Health.

[62] Allen S (2005). International data. J Acquir Immune Defic Syndr (1999)..

[63] King R, Estey J, Allen S, Kegeles S, Wolf W, Valentine C (1995). A family planning intervention to reduce vertical transmission of HIV in Rwanda. AIDS..

[64] USAID (2016). Promoting Integration of Family Planning into HIV and AIDS Programming.

[65] World Health Organization (2009). Strengthening the linkages between family planning and HIV/AIDS policies, programs, and services.

[66] Druce N, Dickinson C, Attawell K, White AC, Standing H (2006). Strengthening linkages for sexual and reproductive health. HIV and AIDS: progress, barriers and opportunities for scaling up.

[67] FHI 360 (2012). Preventing Unintended Pregnancies and HIV.

